# Correction: Atac, A.; Atak, E. The Effect of Stretching Exercises Applied to Caregivers of Children with Development Disabilities on Musculoskeletal Muscle Mobility and Respiratory Function. *Int. J. Environ. Res. Public Health* 2024, *21*, 1361

**DOI:** 10.3390/ijerph23040510

**Published:** 2026-04-16

**Authors:** Amine Atac, Ebrar Atak

**Affiliations:** 1 Department of Physiotherapy and Rehabilitation, Faculty of Health Sciences, Istanbul Gedik University, Istanbul 34876, Turkey; 2 Department of Physiotherapy and Rehabilitation, Faculty of Health Sciences, Yalova University, Yalova 77100, Turkey; ebraratak@hotmail.com

In the original first publication [1], the general information language in the second and third paragraphs of the Introduction section was revised and the expressions in the paragraphs were rearranged because of the coincidental similarity with the publication in the newly added Reference [17]. With this correction, the order of some references has been adjusted accordingly. The revised paragraphs and the newly added reference are shown below.

Adequate hamstring extensibility plays a critical role in enabling functional movement patterns required in everyday life. However, reduced hamstring length is frequently observed in the general population, which may be explained by the muscle’s involvement in sustained postural control and its tendency to remain in a shortened state during prolonged static positions [9,10]. Shortening of the hamstring muscle can alter pelvic alignment by promoting posterior pelvic tilt, which subsequently affects spinal curvatures and pathologies, including a reduction in lumbar lordosis and an increase in thoracic kyphosis. These postural alterations may restrict thoracic mobility, influence diaphragmatic mechanics, and negatively impact respiratory efficiency. Furthermore, hamstring tightness may contribute to dysfunction not only locally but also across anatomically distant segments within the posterior kinetic chain [9,11–14]. The posterior kinetic chain comprises multiple muscle groups, including the spinal extensors, gluteus maximus, hamstring muscles, calf musculature such as the gastrocnemius and soleus, as well as intrinsic muscles of the foot [15]. Muscles forming the posterior chain function collectively to maintain upright posture against gravitational forces. Increased tension in any component of this chain, such as the hamstring muscle, may be transmitted to other interconnected structures. This phenomenon is consistent with the biological tensegrity model, which proposes that bodily tissues operate as an integrated system balancing tensile and compressive forces throughout the body [16–18]. Increased stiffness and reduced flexibility in the hamstring muscles are frequently accompanied by compensatory tension in proximal regions, particularly within the lumbar and shoulder musculature [14].

In the literature, there are studies explaining the relationship between hamstring muscles and respiratory parameters with the myofascial theory [9,16]. According to this theory, the human body is composed of fascia, a single tissue that functions as interconnected chains. Tension in one point of the fascia, which shows integrity, can result in tension or restriction in another part of the body [18]. Previous research has explored the association between hamstring muscle characteristics and respiratory parameters within the framework of myofascial continuity. According to this perspective, fascia functions as a unified connective network, allowing mechanical tension generated in one region to influence distant anatomical areas. In this context, alterations in diaphragmatic mobility or tension may contribute to functional limitations observed during the assessment of hamstring flexibility [15–17].

17. Bırık, B. Hamstring kas Kısalığında Miyofasyal Gevşetme Tekniğinin Posterior Zincir Kaslarının Mobilitesi, Solunum Fonksiyonları, Solunum kas Kuvveti ve Enduransı Üzerine Etkisi. Master’s Thesis, Bezmialem Vakıf University, İstanbul, Turkey, 2018.

The authors state that the scientific conclusions are unaffected. This correction was approved by the Academic Editor. The original publication has also been updated.
